# ChatGPT, et al … Artificial Intelligence, Authorship, and Medical Publishing

**DOI:** 10.1002/acr2.11538

**Published:** 2023-04-10

**Authors:** Daniel H. Solomon, Kelli D. Allen, Patricia Katz, Amr H. Sawalha, Ed Yelin

**Affiliations:** ^1^ Brigham and Women's Hospital, Harvard Medical School; ^2^ University of North Carolina at Chapel Hill, and Durham VA Health Care System Durham North Carolina; ^3^ University of California–San Francisco San Francisco; ^4^ University of Pittsburgh Pittsburgh Pennsylvania

*It seems probable that once the machine thinking method had started, it would not take long to outstrip our feeble powers …. They would be able to converse with each other to sharpen their wits. At some stage, therefore, we should have to expect the machines to take control*.Alan Turing *Intelligent Machinery, A Heretical Theory*

If you have not yet heard of ChatGPT, you will! This artificial intelligence (AI)‐based chatbot is making waves in medicine, education, academic publishing, and more widely. If you are a clinician and dismissed the idea that AI‐powered care is part of our future, think again. AI‐powered chatbots like ChatGPT are being put to the test on clinical scenarios and board examinations and fare pretty well (1). GPT, generative pretrained transformer, describes the next generation in AI‐powered chatbots that not only construct full sentences on topic but now synthesize information from many fields, from many sources, and with tremendous nuance. We tried ChatGPT recently, asking it to create a patient‐facing educational brochure on medications for gout. Almost instantaneously, ChatGPT spit out a brochure that was accurate, written at the correct reading level, and appropriate in its supportive tone.

It is useful to explain a bit more about this type of AI. ChatGPT is just one of several large language model (LLM) interfaces for AI; many vendors are working on other interfaces that will have very similar capabilities. You might already be familiar with narrower forms of AI, which focus on one task, although you may not think of these applications as AI. These tasks might be as narrow as correcting grammar, detecting plagiarism, proof‐reading insurance forms, interpreting radiology imaging, or telling us the weather.

However, with the advent of LLM interfaces, AI has become a co‐author on scientific papers (2). Can an LLM AI tool really co‐author a scientific paper? At this stage, no one doubts that these tools can generate useful text that might accurately synthesize previously collected or original data. But authorship raises other questions about accountability. If the methods that LLM AI tools use to generate text are not transparent (they probably will never be), then who is accountable? One pillar of authorship according to the International Committee of Medical Journal Editors requires that authors agree “to be accountable for all aspects of the work…” (3). At this stage, it is not clear that LLM AI tools can be held accountable, so the American College of Rheumatology (ACR) journal editors and the ACR Committee on Journal Publications have agreed that co‐authorship is not appropriate for these tools (see new Author Instructions; https://onlinelibrary.wiley.com/page/journal/25785745/homepage/guide-to-authors). Another potential issue is that LLM AI tools are trained on existing literature that may be inaccurate and biased. Thus, we also have concerns that unintended biases may be magnified through these tools, often in ways that are not apparent.

We acknowledge that there will likely be many instances when such tools will be used to perform analyses or to contribute to a scientific project. Narrow AI tools are currently widely used in imaging analyses (4). Such contributions should be reported by referring to the specific versions of the tools used by authors and ensuring that the tools are publicly available, even if a fee is required. But, as the Journal of the American Medical Association (JAMA) has appropriately stated, “Authors must take responsibility for the integrity of the content generated by these models and tools” (5).

Some editors have wondered whether LLM AI tools could be used in the peer review process. The ACR journals use AI tools to check for plagiarism and image authenticity. Furthermore, our search tools use narrow AI to find appropriate peer reviewers. However, we have not put AI tools to use as actual “peer reviewers.” Although we do not anticipate substituting human peer reviewers with LLM AI tools, we will monitor whether such tools can be a useful adjunct.

LLM AI tools are not going away and offer great potential for benefits, in clinical care and scientific research. As with almost all innovations, we need to consider the potential negative consequences. For now, LLM AI tools will not be considered for co‐authorship in ACR journals.

Stay tuned…

## AUTHOR CONTRIBUTIONS

All authors were involved in drafting the article or revising it critically for important intellectual content, and all authors approved the final version to be published.

## Supporting information


Disclosure Form
